# Construction and analysis of a network of exercise-induced mitochondria-related non-coding RNA in the regulation of diabetic cardiomyopathy

**DOI:** 10.1371/journal.pone.0297848

**Published:** 2024-03-28

**Authors:** Shuo Wang, Jiacong Li, Yungang Zhao

**Affiliations:** Tianjin Key Laboratory of Exercise Physiology and Sports Medicine, Tianjin University of Sport, Tianjin, China; Universita degli Studi della Campania Luigi Vanvitelli, ITALY

## Abstract

Diabetic cardiomyopathy (DCM) is a major factor in the development of heart failure. Mitochondria play a crucial role in regulating insulin resistance, oxidative stress, and inflammation, which affect the progression of DCM. Regular exercise can induce altered non-coding RNA (ncRNA) expression, which subsequently affects gene expression and protein function. The mechanism of exercise-induced mitochondrial-related non-coding RNA network in the regulation of DCM remains unclear. This study seeks to construct an innovative exercise-induced mitochondrial-related ncRNA network. Bioinformatic analysis of RNA sequencing data from an exercise rat model identified 144 differentially expressed long non-coding RNA (lncRNA) with cutoff criteria of *p<* 0.05 and fold change ≥1.0. GSE6880 and GSE4745 were the differentially expressed mRNAs from the left ventricle of DCM rat that downloaded from the GEO database. Combined with the differentially expressed mRNA and MitoCarta 3.0 dataset, the mitochondrial located gene Pdk4 was identified as a target gene. The miRNA prediction analysis using miRanda and TargetScan confirmed that 5 miRNAs have potential to interact with the 144 lncRNA. The novel lncRNA-miRNA-Pdk4 network was constructed for the first time. According to the functional protein association network, the newly created exercise-induced ncRNA network may serve as a promising diagnostic marker and therapeutic target, providing a fresh perspective to understand the molecular mechanism of different exercise types for the prevention and treatment of diabetic cardiomyopathy.

## Introduction

Diabetic cardiomyopathy (DCM) is a pathophysiological condition stemming from diabetes mellitus that can lead to heart failure [1]. The World Health Organization (WHO) reports that approximately 463 million people worldwide have diabetes, constituting 9.3% of the global population. In China, diabetes patients have surged from 0.7% in 1980 to 12.8% in 2017, marking an 18-fold increase over forty years [2]. Furthermore, over a third of Chinese adults have pre-diabetes with inadequate awareness, treatment, and management rates [3]. Diabetic cardiomyopathy significantly increases the risk of heart failure, cardiovascular events, and mortality among individuals with diabetes [1, 4]. Effective preclinical diagnosis is crucial in preventing the development of diabetic cardiomyopathy. Echocardiography remains the cornerstone for diagnosing diabetic cardiomyopathy [5]. Cardiac magnetic resonance imaging (MRI) has gained prominence as a non-invasive modality for assessing cardiac morphology, function, and tissue characterization. Novel biomarkers have also emerged as potential diagnostic tools. Recent studies have focused on evaluating circulating microRNAs and long non-coding RNA, cardiac troponins, and other inflammatory markers as indicators of cardiac damage and dysfunction in diabetic cardiomyopathy [6, 7]. It is important to mention that the diagnosis of diabetic cardiomyopathy remains challenging, requiring the exclusion of other causes of cardiomyopathy and careful clinical evaluation.

The development of DCM is closely associated with insulin resistance, inflammation, and oxidative stress, with a significant contribution from mitochondria [8]. Elevated levels of reactive oxygen species (ROS) cause increased oxidative stress and are related to dysfunctions in myocardial contraction and relaxation and mitochondrial damage. High levels of ROS can cause damage to cellular DNA, proteins, and lipids which results in irreversible cell damage and death, ultimately leading to myocardial dysfunction. The main cause of oxidative stress in dilated cardiomyopathy is the increased amount of ROS from mitochondria [9]. Insulin resistance decreases glucose utilization and, when combined with abnormal fatty acid metabolism and elevated ROS levels, provokes damage to mitochondrial proteins and deregulated fusion and fission, resulting in amplified mitochondrial fission and eventual mitochondrial dysfunction [10]. In type 2 diabetes, insulin resistance damages mitochondrial autophagy, leading to decreased effectiveness in clearing damaged mitochondria and exacerbating apoptosis [11, 12]. High glucose treatment of cardiomyocytes results in reduced expression of the mitochondrial calcium transporter (MCU) and a subsequent decrease in mitochondrial matrix-free calcium levels. This leads to decreased activity of the pyruvate dehydrogenase complex, lowered glucose oxidation-reduction, increased fatty acid oxidation, decreased mitochondrial membrane potential, increased oxidative stress, and ultimately, increased apoptosis of cardiomyocytes [1]. Previous studies have demonstrated a correlation between heightened pyruvate dehydrogenase kinase 4 (PDK4) expression in diabetic rats and reduced cardiac function. This upregulation of PDK4 leads to increased fatty acid oxidation, potentially resulting in mitochondrial and cardiac dysfunction [13, 14].

Regular physical activity can positively impact preventing and managing diabetes by enhancing glucose and insulin metabolism and decreasing the likelihood of cardiovascular disease. Exercise can also improve cardiovascular and pulmonary health, leading to decreased incidence and mortality of DCM [15]. Acute or chronic exercise can benefit both healthy and diabetic populations. This intervention improves important functions such as insulin action, body weight, glucose tolerance, GLUT-4 response, IRS1 and Akt phosphorylation, inflammatory response, mitochondrial biogenesis, oxidative capacity, mitochondrial enzyme activity, ATP synthesis level, and others [16, 17]. Aerobic exercises, either alone or in combination with resistance training, offer potential anti-atherosclerotic benefits, a reduced risk of cardiovascular disease, and lowered triglyceride levels in the liver [18]. High-intensity interval training has the potential to reduce liver fat levels, alkaline phosphatase, and glycosylated hemoglobin levels [19]. In contrast, aerobic exercise can improve insulin sensitivity, whereas resistance training can increase muscle mass and GLUT4 expression, thereby enhancing blood glucose intake [20]. Exercise training induced changes in exerkine concentrations that have been shown to mediate type 2 diabetes and may be relevant to metabolic control in type 2 diabetes mellitus patients [21]. Meta-analysis indicated that physical exercise training could induces an increase in adiponectin, FGF-21, and IL-10 and a decrease in fetuin-A, IL-6, leptin, resistin, TNF-a, visfatin and also HbA1c [21]. Skeletal muscle, an important myokine secretion organ, plays a crucial role in mediating diabetes and diabetic cardiomyopathy through various metabolic pathways via muscle-organ crosstalk [22].

Non-coding RNA (ncRNA) is found to be overexpressed in individuals with diabetes and plays a critical role in regulating the functioning of the pancreas, liver, skeletal muscle, and adipose tissue during the onset of diabetes. This discovery highlights its potential as a biomarker for diagnosing and treating diabetes [23, 24]. Long non-coding RNA (lncRNA) plays a crucial role in regulating the cell cycle, cell differentiation, cell metabolism, and various pathological changes by influencing factors such as gene transcription, translation, and RNA stability [25, 26]. Furthermore, lncRNA regulates mitochondrial protein homeostasis, affecting mitochondrial quality control and aiding in the management of diabetes and related complications [27]. Moderate and high-intensity exercise can prevent diabetes-induced heart and coronary artery dysfunction. Additionally, miR-126 has been identified as a potential diagnostic biomarker for detecting early coronary artery vascular abnormalities and tracking dynamic changes in coronary artery health due to exercise [28].

Therefore, this study aims to investigate the regulatory mechanism of exercise-induced mitochondrial-related lncRNA network in improving diabetic cardiomyopathy. Moreover, it aims to identify the interrelation between differential genes induced by exercise in skeletal muscle and the regulation of cardiac function. Additionally, this study aims to discover novel lncRNA biomarkers that can offer new insights into the diagnosis and treatment of diabetic cardiomyopathy.

## Materials and methods

### Animals and exercise training

Eighteen healthy male SD rats (6 weeks old) were purchased from Beijing Vital River Laboratory Animal Technology Company. The rats were kept in a standard environment with free access to food and water and subjected to a 12-hour light/dark cycle. The temperature of their environment was maintained at 18–22°C with a relative humidity of 50%-60%. The study was approved by the Ethical Committee of Tianjin University of Sport (20190311). The rats were divided into three groups at random (6 for each group): the control group (C), the long-term aerobic exercise group (OE), and the acute exhaustive exercise group (AE). The OE group engaged in aerobic exercise on a treadmill inclined at 0° for 60 minutes per day, five days a week, at a speed of 20 m/min for eight weeks. In the AE group, rats underwent a single bout of acute exhaustive exercise following eight weeks of normal feeding. The rats ran on a treadmill at a speed of 10m/min and a slope of 0°, enduring an incremental increase in load. The speed increased by 5m/min every 5 minutes until exhaustion. The rats in control were inactivity. The rats were anesthetized with isoflurane and bled to death from the heart, after which their gastrocnemius muscle was extracted.

### RNA extraction and sequencing analysis

Three samples were selected randomly and the total RNA was extracted from the gastrocnemius muscle samples using RNA isolation kit (Vazyme, Nanjing, China) according to the manufacturer’s protocol. The raw reads were generated by high-throughput sequencing and show in the FASTQ format. In order to obtain high-quality reads for subsequent analysis, the raw reads were subjected to a quality filter. Trimmomatic [29] was used to filter the low-quality bases and N-bases or low-quality reads. HISAT2 [30] was used to align clean reads to the reference genome of the experimental species, the sample was assessed by genomic and gene alignment. And Stringtie [31] was utilized to assemble and merged the transcripts. Four computational approaches include CPC2/CNCI/Pfam/CPAT were combined to sort non-protein coding RNA candidates. StringTie was used to calculate FPKMs of both lncRNA and coding genes in each sample. Gene FPKMs were computed by summing the FPKMs of transcripts in each gene group. The differential transcripts with *p*-values < 0.05 and fold change ≥1 were set as the threshold for significantly differential expression of the differentially expressed genes (DEGs). The topGO R packages and KOBAS [32] software was used to make Gene Ontology (GO) enrichment and Kyoto Encyclopedia of Genes and Genomes (KEGG) pathway enrichment analysis. Miranda (http://mirtoolsgallery.tech/mirtoolsgallery/) and TargetScan (https://www.targetscan.org) were used to predict the miRNAs that interaction with differently expressed mRNA and lncRNA. The predicted miRNAs in both the two software were filtered out for predictive analysis. Gene interaction and enrichment analysis were conducted with the help of STRING (https://string-db.org/).

### Identification of differentially expressed genes from GEO database

"Diabetic cardiomyopathy" was served as the search term on the GEO database (https://www.ncbi.nlm.nih.gov/geo/), and two gene datasets, GSE6880 and GSE4745, were acquired from the database. The GSE6880 dataset includes microarray results from six left ventricular myocardial rat samples (three in the control group and three in the diabetes group). GSE4745 contained microarray results from 24 left ventricular myocardial rat samples. Eight samples from both the control and diabetes groups on day 42 were analyzed here for subsequent investigation. GEO2R was used to filter differentially expressed mRNAs from GSE6880 and GSE4745. A fold change of ≥ 1.0 and a *P* value of < 0.05 were set as the cutoff criteria. Then the expressed differently mRNAs in all the two datasets and the sequencing results were filtered out as DEGs for further analysis.

### Identification of differentially expressed mitochondrial protein-encoding genes and construction of ncRNA network

The roster of mitochondrial genes was obtained from the MitoCarta 3.0 database (https://www.broadinstitute.org/). In order to identify the differentially expressed mitochondrial protein-coding genes relevant to diabetic cardiomyopathy, the commonly expressed genes across MitoCarta 3.0, sequencing data, GSE6880 and GSE4745 were filtered out. The functional protein association network was visualized by STRING (https://cn.string-db.org/). The results of the miRNA prediction allowed for the construction of miRNA-mRNA and miRNA-lncRNA. An exercise-induced regulatory network involving mitochondria-related lncRNA-miRNA-mRNA was established and visualized with Cytoscape 3.9.1 (http://cytoscape.org/).

## Results

### Differentially expressed lncRNA and mRNA after different exercise training

**Fig 1:**
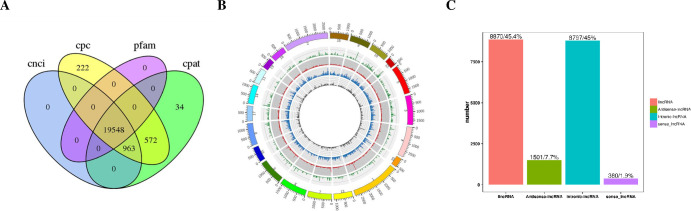
lncRNA prediction results. A. Venn of predicted lncRNA. B. Ringlike of the distribution of lncRNA on the chromosome, in which the outermost layer is the chromosomal ring of the genome, and the sense strand lncRNA ring (green), intergenic zone lncRNA ring (red), antisense lncRNA ring (grey) and intron LNC RNA ring (blue) are shown in sequence from outside to inside; C. Histogram of lncRNA classification.

**Fig 2:**
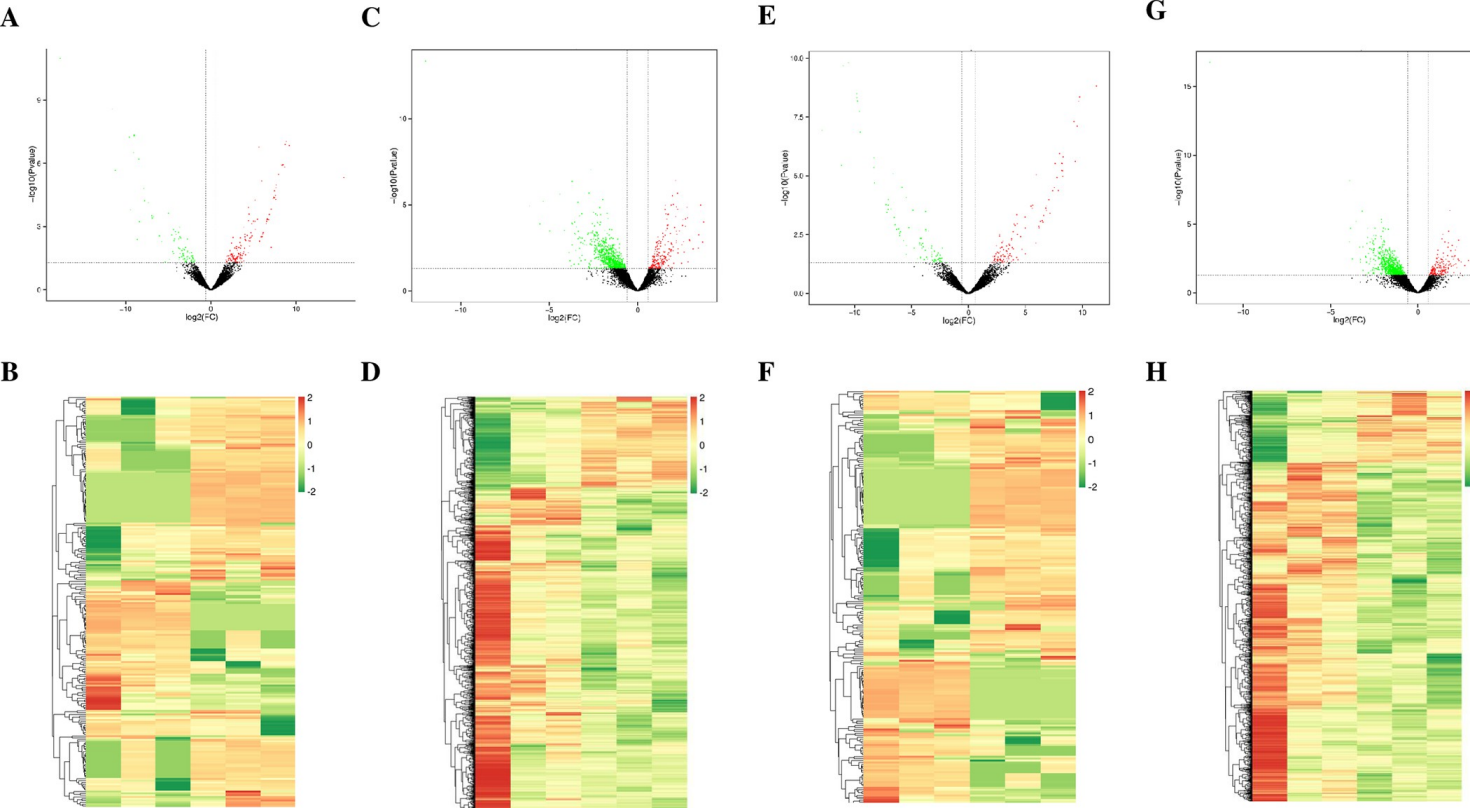
Results of differentially expressed lncRNA and mRNA. A, B. Volcano and heatmap results of long-term aerobic exercise-induced differential expression of lncRNA; C, D. Volcano and heatmap results of differentially expressed mRNA induced by long-term aerobic exercise; E, F. Volcano and heatmap results of differentially expressed lncRNA induced by a single acute exercise; G, H. Volcano and heatmap results of differentially expressed mRNA induced by a single acute exercise.

**Fig 3:**
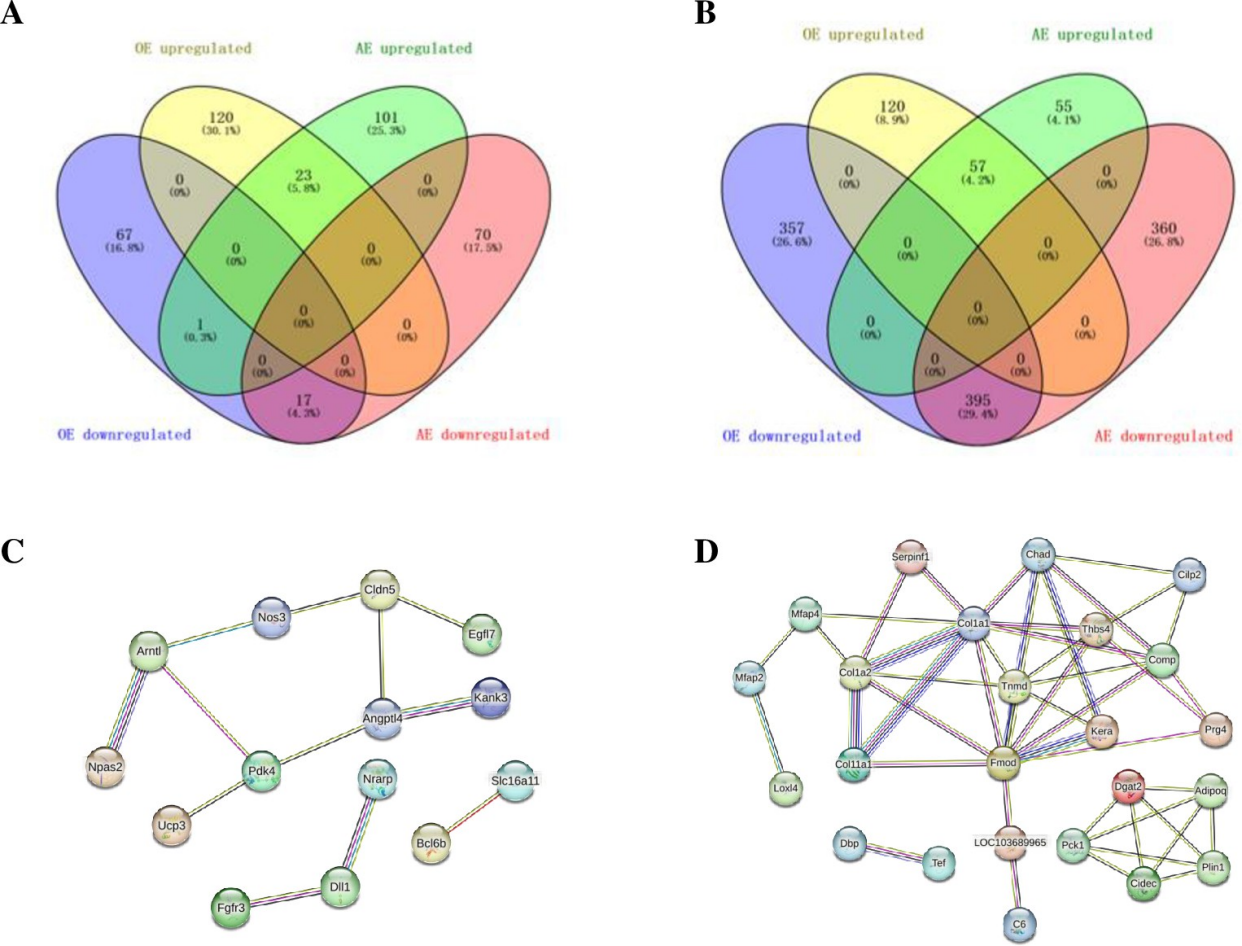
Statistics and interaction analysis of differentially expressed lncRNA and mRNA. A. Venn of long-term aerobic exercise and a single acute exercise-induced differential expression of lncRNA; B. Venn of differentially expressed mRNA induced by long-term aerobic exercise and single acute exercise; C. Gene interaction network of exercise-induced co-upregulated genes; C. Gene interaction network of exercise-induced co-downregulated genes.

A total of 19,548 long non-coding RNAs (lncRNA) were obtained from the sequencing results (Fig 1A). The distribution and chromosomal classification of the lncRNA are presented in Fig 1B and 1C. Differentially expressed lncRNA and messenger RNAs (mRNAs) were confirmed with the cutoff criterion of [log2 (fold-change)] ≥1.0, and *P* < 0.05. Fig 2 displays the analysis results of differentially expressed lncRNA and mRNAs. As illustrated in Fig 3A, the OE group had 228 differentially expressed lncRNA, with 143 being upregulated (including 124 newly discovered) and 85 being downregulated (including 78 newly discovered), compared to the control group. The AE group, on the other hand, had 212 differentially expressed lncRNA, of which 125 were upregulated (including 108 novel) and 87 were downregulated (including 81 novel). As illustrated in Fig 3B, 929 mRNA were differentially expressed in the OE group, comprising 177 upregulated genes (15 of which were novel) and 752 downregulated genes (535 of which were novel). Similarly, in the AE group, 867 mRNA were differentially expressed, with 112 upregulated genes (including 27 novel genes) and 755 downregulated genes (including 652 novel genes). The interaction relationship between the overlapped genes is displayed in Fig 3C and 3D.

**Fig 4:**
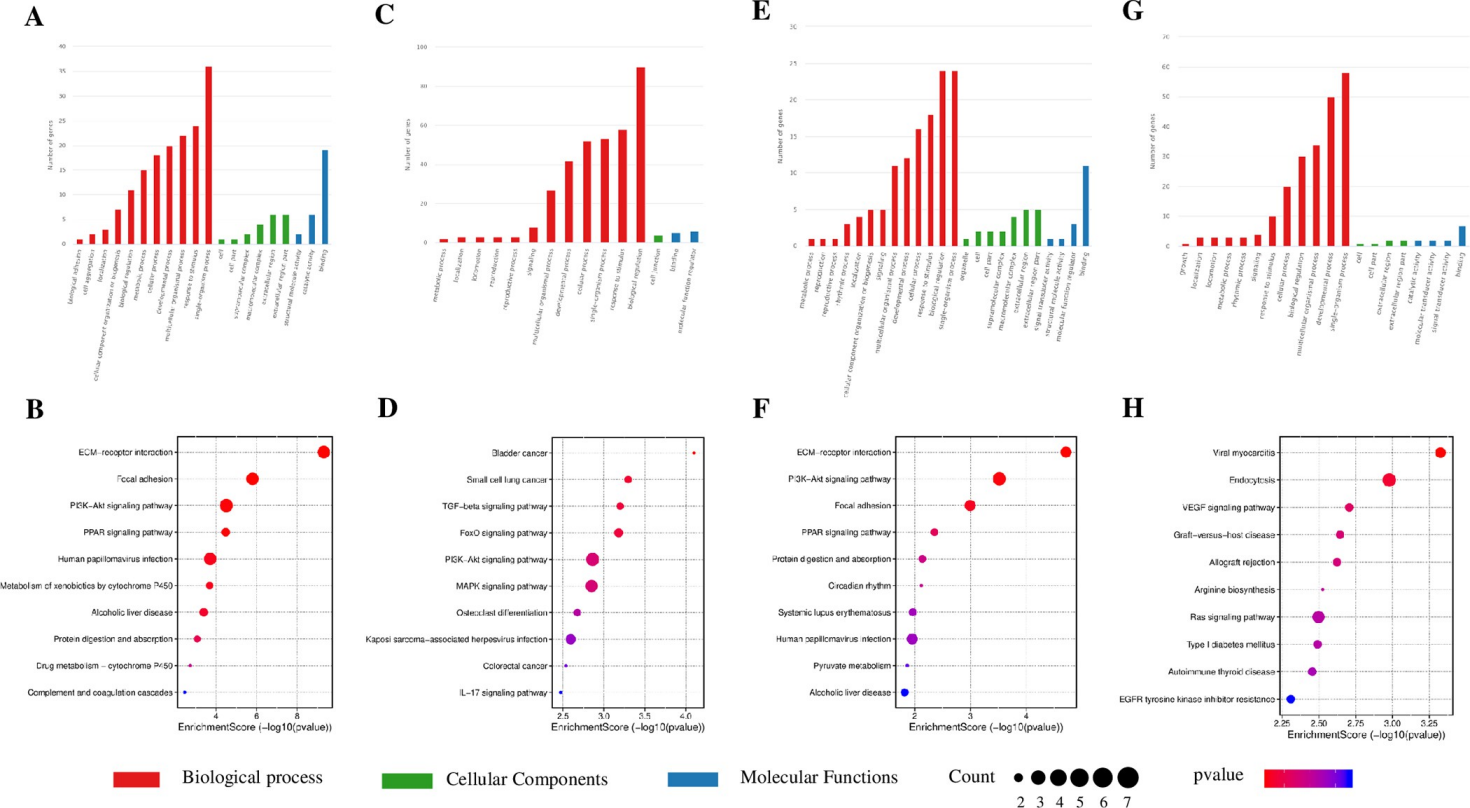
Results of functional enrichment and pathway analysis of differentially expressed mRNA. A, B. GO enrichment (A) and KEGG pathway (B) analysis of differentially expressed upregulated genes induced by long-term aerobic exercise; C, D. GO enrichment (C) and KEGG pathway (D) analysis of differentially expressed downregulated genes induced by long-term aerobic exercise; E, F. GO enrichment (E) and KEGG pathway (F) analysis of differentially expressed upregulated genes induced by a single acute exercise; G, H. GO enrichment (G) and KEGG pathway (H) analysis of differentially expressed downregulated genes induced by a single acute exercise.

The functional enrichment analysis of differentially expressed genes is presented in Fig 4. In the OE group, 119 terms were associated with upregulated mRNAs, including 83 terms in the biological process network, 9 in the cellular component networks, and 27 in molecular functions (Fig 4A). Similarly, 119 terms were linked to downregulated mRNAs, comprising of 83 terms in the biological process network, 9 in the cellular component networks, and 27 in molecular functions (Fig 4C). In the AE group, 87 terms were associated with upregulated mRNAs, including 64 in the biological process network, 9 in cellular component networks, and 14 in molecular functions indicating molecular functions (Fig 4E). In contrast, 104 terms were linked to down-regulated mRNAs, with 92 in the biological process network, 3 in cellular component networks, and 9 in molecular functions (see Fig 4G).

The KEGG pathway analysis depicted in Fig 4 shows differential gene expression. In the OE group, the PI3K-Akt signaling pathway, ECM-receptor interaction, and human papillomavirus infection had upregulated genes (Fig 4B). In contrast, the PI3K-Akt signaling pathway, MAPK signaling pathway and Kaposi sarcoma-associated herpesvirus infection had downregulated genes (Fig 4D). In the AE group, the genes that were upregulated were predominantly enriched in the PI3K-Akt signaling pathway, ECM-receptor interaction, and human papillomavirus infection, as shown in Fig 4F. Conversely, the genes that were downregulated were primarily enriched in the endocytosis, Ras signaling pathway and viral myocarditis, as shown in Fig 4H.

### Identification of differentially expressed genes related to diabetic cardiomyopathy

**Fig 5:**
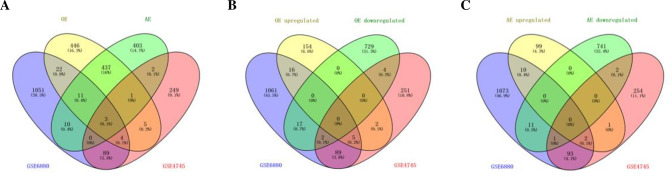
Differentially expressed genes in skeletal muscle associated with diabetic cardiomyopathy. A. Venn of differentially expressed genes related to diabetic cardiomyopathy induced by long-term aerobic exercise and acute exercise; B, C. Venn of differentially expressed genes related to diabetic cardiomyopathy induced by long-term aerobic exercise (B) and single acute exercise (C).

To identify differentially expressed genes related to DCM in skeletal muscle after exercise, we downloaded the GSE6880 and GSE4745 datasets and identified overlapping genes (Fig 5A). A total of 46 genes were found to be differentially expressed after aerobic exercise, with 23 up-regulated and 23 down-regulated (Fig 5B). Twenty-seven genes related to diabetic cardiomyopathy were differentially expressed in the AE group, with 13 upregulated and 14 downregulated (Fig 5C). After the two exercise models, three genes related to diabetic cardiomyopathy overlapped: Col11a1, Pdk4, and Comp. Col11a1 and Comp were upregulated, while Pdk4 was downregulated. These results showed that there is a degree of consistency between single sessions of acute and prolonged aerobic exercise in regulating gene expression. However, prolonged aerobic exercise has a more significant impact on gene expression. Understanding the molecular changes caused by exercise is crucial in selecting appropriate exercise interventions for diabetic cardiomyopathy.

### Identification of differentially expressed genes encoding mitochondrial proteins associated with diabetic cardiomyopathy

**Fig 6:**
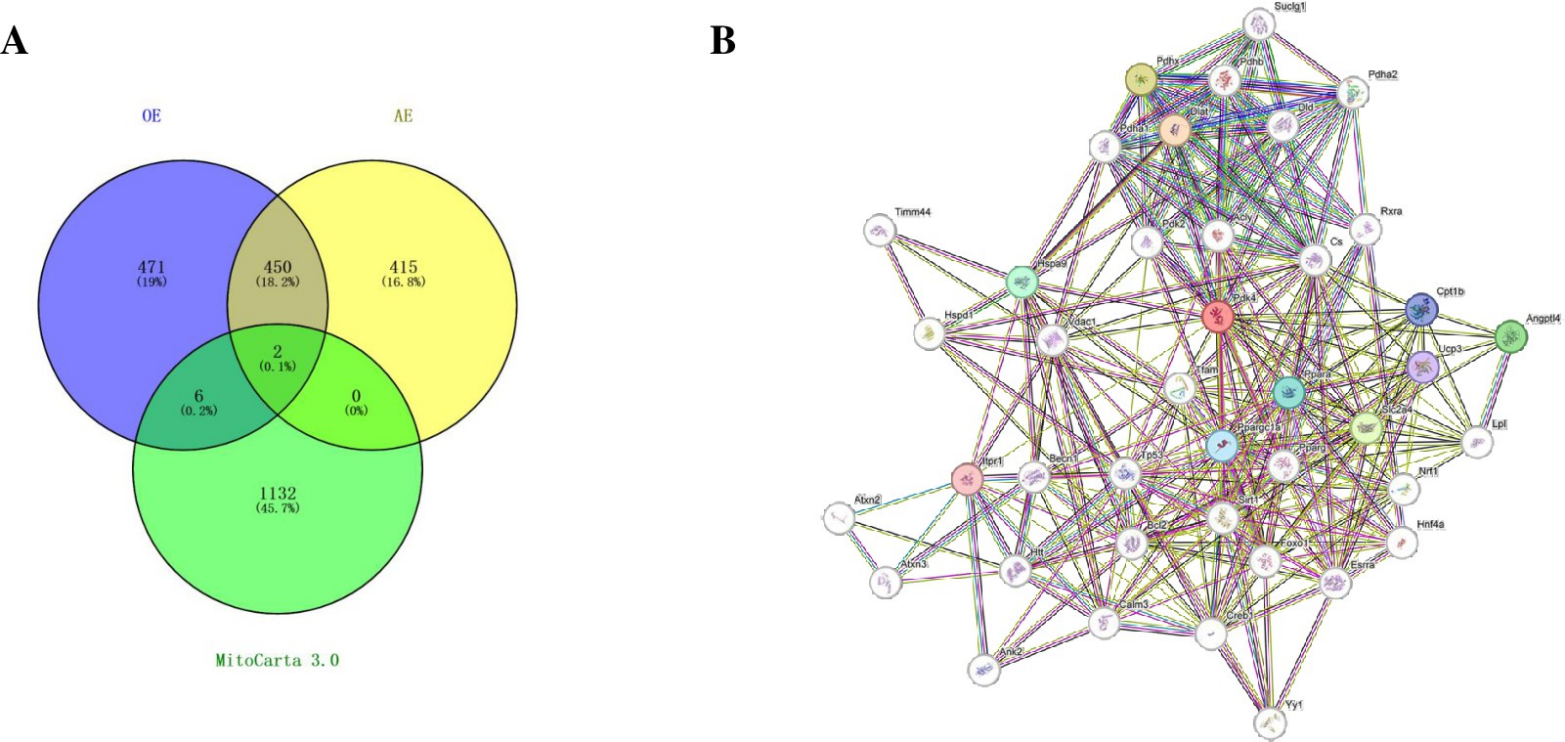
Differentially expressed genes encoding mitochondrial proteins associated with diabetic cardiomyopathy. A. Venn of differentially expressed genes and mitochondrial-related genes induced by long-term aerobic exercise and single acute exercise; B. Functional protein interaction network of Pdk4. The red node represents Pdk4.

The mitochondrial gene set, which consists of 1140 protein-coding genes, was downloaded from the MitoCarta3.0 database. Pdk4 and Ucp3 were filtered out as the DEGs in the OE group, AE group, and MitoCarta 3.0 mitochondrial gene set. Both of these genes were downregulated after the two exercise models (Fig 6A). Combined with genes associated with diabetic cardiomyopathy, Pdk4 was validated as the target gene that may play a key role in mediating diabetic cardiomyopathy during exercise. The protein association network revealed that Pdk4 is involved in various pathways, including energy metabolism, mitochondrial gene expression, and signaling transduction (Fig 6B). According to the association network, Pdk4 may regulate pyruvate and fatty acid metabolism through interactions with Pdk2, Pdha1/2, Ppara, Pparg, and Slc2a4 [33–35]. Additionally, Pdk4 may interact with Vdac1 to regulate mitochondrial calcium, influencing metabolic balance and autophagy [36, 37]. These associations demonstrate that Pdk4 plays a crucial role in improving mitochondrial function and cardiovascular disease through mediating oxidation metabolism, autophagy, oxidative stress, and mitochondrial dynamics. This provides evidence of the molecular impact of exercise on the improvement of cardiovascular health and highlights the crosstalk between the skeletal muscle and the myocardium during exercise.

### Construction and analysis of mitochondrial-related non-coding RNA network in diabetic cardiomyopathy

**Fig 7:**
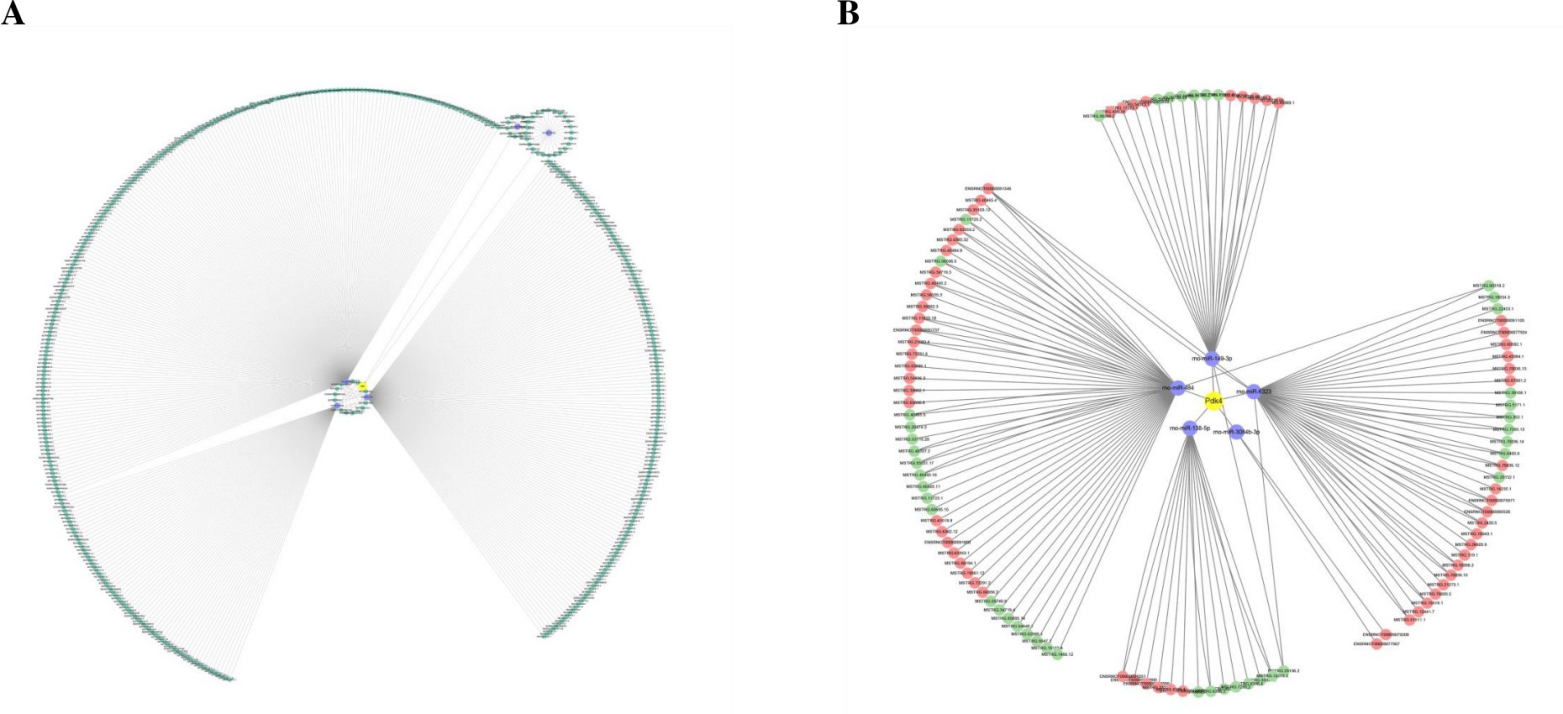
Construction of mitochondrial-related lncRNA-miRNA-mRNA regulatory network. A. Mitochondria-related lncRNA-miRNA-mRNA network. The yellow node in the figure represents Pdk4, the blue node represents miRNAs, and the dark green node represents lncRNA. B. Exercise-induced differential expression lncRNA-miRNA-Pdk4 network. In the figure, the yellow node represents Pdk4, the blue node represents miRNAs, the red node represents upregulated lncRNA, and the green node represents downregulated lncRNA.

Based on the miRNA prediction results, 443 lncRNA have the potential to interact with five miRNAs (miR-138-5p, miR-149-3p, miR-484, miR-3084b-3p, miR-6323), which collectively target Pdk4 (Fig 7A). Out of these, 144 lncRNA with different expressions were filtered out, and 70 of them showed differential expression after long-term aerobic exercise (43 upregulated and 27 downregulated). Additionally, 74 lncRNA showed differential expression following a single acute exercise session (44 were upregulated and 30 downregulated). It was found that 16 lncRNA interact with miR138-5p, 22 lncRNA interact with miR-149-3p, 59 lncRNA interact with miR-484, 2 lncRNA interact with miR-3084b-3p, and 45 lncRNA interact with miR-6323. Consequently, a total of 144 novel regulatory pathways involving lncRNA, microRNAs, and Pdk4 were constructed (Fig 7B). These findings suggest that extended periods of aerobic exercise may regulate the progression of diabetic cardiomyopathy by influencing the expression of lncRNA that target mitochondrial protein and impact mitochondrial function through various pathways.

## Discussion

For heart disease patients without diabetes, medication has a beneficial therapeutic impact. Empagliflozin is an effective treatment for attenuating myocardial toxicity caused by Doxorubicin therapy and enhancing both cardiomyocyte and mitochondrial function [38]. While for diabetic cardiomyopathy, which ranks among the primary causes of heart failure in diabetes patients, exercise exhibits insightful effects in improving cardiovascular health in diabetes. Regular exercise bears a substantial effect on improving conditions such as obesity and diabetes. It has become an efficacious way of treating diabetic cardiomyopathy. Regular exercise plays a crucial role in reducing the likelihood of cardiovascular disease by enhancing glucose and insulin metabolism [19]. Endurance exercise induces significant changes in the expression of 71 proteins in human skeletal muscle, primarily promoting oxidative phosphorylation and the expression of tricarboxylic acid cycle-related proteins, while attenuating substrate utilization-related proteins [39]. Although numerous studies indicate that exercise improves molecular changes in the diabetic heart, few reports establish links between molecular changes in skeletal muscle and the myocardium. During exercise, skeletal muscle constitutes a crucial metabolic and motor organ that delivers the primary blood and nutrient supply to the body. It continuously metabolizes and secretes numerous biological molecules into the bloodstream that affect different tissues and organ systems. Some of these molecules, such as IL-6, Irisin, and IGF1 [40, 41], perform important regulatory functions. In this study, a comparison was made between the effects of long-term aerobic exercise and single acute exercise on lncRNA and gene expression in skeletal muscle. The results showed that 929 and 867 genes were differentially expressed, respectively, with 58 of them being related to diabetic cardiomyopathy. These findings confirm that exercise-induced molecular adaptation in skeletal muscle may play a crucial role in regulating cardiac function. Muscle-heart crosstalk is a crucial mechanism in comprehending the role of exercise in treating cardiovascular disease. It has been found that various types of exercise may regulate this pathway through distinct molecular pathways.

Exercise can induce changes in the expression of lncRNA and miRNA, ameliorating diabetes and lesions related to diabetic cardiomyopathy through various pathways. Several differentially expressed lncRNA and miRNA may offer potential effective targets for diagnosing and treating diabetic cardiomyopathy [28, 42, 43]. The study found that long-term aerobic exercise resulted in changes to the expression of 228 lncRNA in skeletal muscle, and 7005 target genes were predicted, with 333 genes linked to diabetic cardiomyopathy. Additionally, a single acute exercise resulted in changes to the expression of 212 lncRNA in skeletal muscle, and 5718 target genes were predicted. Among the genes that exhibited differential expression, 58 were linked to diabetic cardiomyopathy while eight genes were linked to mitochondria, specifically the gene encoding for the mitochondrial protein pyruvate dehydrogenase–PDK4. PDK4 is a key regulator of glucose metabolism, fatty acid oxidation, and oxidation-reduction. It was found PDK4 expression plays a key role in regulating glucose metabolism in aging and therefore mediating cardiomyocyte injury in ischemia reperfusion and aging [44, 45]. PDK4 is largely responsible for inhibiting pyruvate dehydrogenase (PDH) in the presence of fatty acids and increasing the reliance of the heart on fatty acid oxidation for energy production [46, 47]. PDK4 deletion resulted in an increase in pyruvate dehydrogenase activity and consequently an increase in glucose relative to fatty-acid oxidation [48]. PDK4 can also inhibit ferroptosis by blocking pyruvate dehydrogenase-dependent pyruvate oxidation [35]. In cardiovascular disease, pyruvate dehydrogenase kinase (PDK)-dependent inhibition of PDH axis is a major immunometabolic pathway associated with vascular inflammation, targeting this axis could inhibit vascular inflammation and atherogenesis [49]. Exercise is thought to cause significant adaptations in skeletal muscle lncRNA and gene expression. Differentially expressed lncRNA may impact mitochondrial function through various pathways. Therefore, it may regulate the function of the heart in individuals with diabetes.

The buildup of free fatty acids in the hearts of diabetic patients, along with a reduction in glucose uptake mediated by insulin, results in heightened cardiac oxygen consumption and mitochondrial dysfunction, ultimately leading to cell death of myocytes and impairment in ventricular function [50]. Studies show that PDK4 expression is heightened in calcified blood vessels of atherosclerosis patients. This can compromise the integrity of the mitochondrial-related endoplasmic reticulum membrane, impair mitochondrial respiratory capacity, encourage glycolysis metabolism, and limit lysosomal degradation by inhibiting V-ATP enzyme and LDHB interaction. Ultimately, this leads to reduced autophagy activity and vascular calcification [37]. However, right ventricular hypertrophy stimulation activates PDK4, promoting glycolysis and causing a decline in right ventricular function [51]. Myocardial ischemia-reperfusion injury can be alleviated through overexpression of miR-148, ultimately improving myocardial antioxidant levels, alleviating myocardial apoptosis and immune dysfunction, and mitigating myocardial dysfunction via PDK4 inhibition [52]. PDK4 can interact with Hmgcs2, promoting its expression and resulting in cardiomyocyte apoptosis and myocardial injury [53]. The mitochondria-associated endoplasmic reticulum membrane (MAM) is a structural link between mitochondria and endoplasmic reticulum (ER). PDK4 interacts with and stabilizes the IP3R1-GRP75-VDAC1 complex at the MAM interface. Inhibiting PDK4 could reduce MAM formation and improve insulin signaling by preventing MAM-induced mitochondrial Ca^2+^ accumulation, mitochondrial dysfunction, and ER stress [54].

The miR-138-5p has been demonstrated to play important role in regulating myocardial infarction, cardiomyocyte pyroptosis and apoptosis, myocardial ischemia/reperfusion injury via various pathways [55–59]. The miR-149-3p was elevated in the mouse heart after whole-body irradiation for 48 hours [60]. The miR-484 could directly targete mRNA of Yap1 to inhibit cell viability, and promote apoptosis and inflammation in LPS-treated H9c2 cells [61]. While the regulation mechanism of these miRNAs with PDK4 in diabetic cardiomyopathy remains unclear, and the effect of miR-3084b-3p and miR-6323 in cardiovascular disease has yet to be reported. It is believed that these interesting findings may provide new insights on diabetic cardiomyopathy via the novel ncRNA-PDK4 axis through mediating mitochondrial quality control, oxidation metabolism, oxidative stress and inflammation. Consequently, investigating the mechanism of the novel lncRNA-miRNA-PDK4 network may serve as a promising diagnostic marker and therapeutic target, providing a fresh perspective to understand the molecular mechanism of different exercise types for the prevention and treatment of diabetic cardiomyopathy.

## Conclusion

Exploring exercise-induced molecular changes can aid in understanding the mechanisms by which exercise combats cardiovascular disease. The newly constructed ncRNA network of mitochondrial-related lncRNA-miRNA-PDK4 is expected to provide a promising diagnostic marker and therapeutic target, offering a new preventive and diagnostic strategy for diabetic cardiomyopathy.

## Supporting information

S1 Raw data The raw sequencing data of the rats. The control group is represented by GSE31, GSE34, and GSE35, while GSE21, GSE24, and GSE25 represent the acute exhaustive exercise group and GSE11, GSE12, and GSE13 represent the long-term aerobic exercise group.
(XLSX)

S1 Data GSE6880 was downloaded from the GEO database and represents the sequencing of eight-week-old rats that were injected with streptozotocin or buffer alone at the age of eight weeks. The heart was obtained at 12 weeks, which means that the animals were diabetic for four weeks. The left ventricle of the heart was analyzed. 3 for each group.
(XLSX)

S2 Data GSE4745 was obtained from the GEO database, which includes data on 150g male Wistar rats (Harlan) that were administered 65 mg/kg of streptozotocin to induce Type 1 diabetes. Four replicates of control and diabetic rat ventricles were removed and frozen at three time points for total RNA isolation and hybridization to the Affymetrix RG-U34A microarray. The analysis in this article focused on eight samples collected at 42 days, with four samples for each group.
(XLSX)
